# Specific up-regulation of p21 by a small active RNA sequence suppresses human colorectal cancer growth

**DOI:** 10.18632/oncotarget.15918

**Published:** 2017-03-06

**Authors:** Lu-Lu Wang, Hui-Hui Guo, Yun Zhan, Chen-Lin Feng, Shuai Huang, Yan-Xing Han, Wen-Sheng Zheng, Jian-Dong Jiang

**Affiliations:** ^1^ State Key Laboratory of Bioactive Substance and Function of Natural Medicines, Institute of Materia Medica, Chinese Academy of Medical Sciences and Peking Union Medical College, Beijing 100050, People’s Republic of China

**Keywords:** p21-saRNA-322, small active RNA (saRNA), colorectal cancer, tumor growth suppression, xenograft tumor mode

## Abstract

The double stranded small active RNA (saRNA)- p21-saRNA-322 inhibits tumor growth by stimulating the p21 gene expression. We focused our research of p21-saRNA-322 on colorectal cancer because 1) p21 down-regulation is a signature abnormality of the cancer, and 2) colorectal cancer might be a suitable target for *in situ* p21-saRNA-322 delivery. The goal of the present study is to learn the activity of p21-saRNA-322 in colorectal cancer. Three human colorectal cancer cell lines, HCT-116, HCT-116 (p53–/−) and HT-29 were transfected with the p21-saRNA-322. The expression of P21 protein and p21 mRNA were measured using the Western blot and reverse transcriptase polymerase chain reaction (RT-PCR). The effect of p21-saRNA-322 on cancer cells was evaluated *in vitro*; and furthermore, a xenograft colorectal tumor mode in mice was established to estimate the tumor suppressing ability of p21-saRNA-322 *in vivo*. The results showed that in all three colorectal cancer cell lines, the expression of p21 mRNA and P21 protein were dramatically elevated after p21-saRNA-322 transfection. Transfection of p21-saRNA-322 caused apoptosis and cell cycle arrest at the G_0_/G_1_. Furthermore, anti-proliferation effect, reduction of colonies formation and cell senescence were observed in p21-saRNA-322 treated cells. Animal studies showed that p21-saRNA-322 treatment significantly inhibited the HT-29 tumor growth and facilitated p21 activation *in vivo*. These results indicated that, p21-saRNA-322-induceded up-regulation of p21 might be a promising therapeutic option for the treatment of colorectal cancer.

## INTRODUCTION

Colorectal cancer (CRC) is the third most commonly diagnosed cancer and the third leading cause of cancer death worldwide [1]. Despite the rapid advancement in diagnosis and therapy for CRC, conventional cytotoxic chemotherapy such as 5-fluorouracil based adjuvant remains to be the necessary approach for the treatment. Severe side effect coupled with high incidents of drug resistance is still the main challenge, which urgently reinforce the requirements for finding alternative way to deal with this problem [2].

P21, a multifunctional cyclin-dependent kinases (CDK) inhibitor (CKI), is a downstream protein of P53. During the responses to DNA damage, P53 was induced and specifically bound its consensus binding sequences in the regulatory region of p21 (wild-type p53-activated fragment) to trans-activate the genes, causing prevention of cell cycle progression via arresting cells in the G1/s [3, 4]. In addition to inhibiting CDK activity, P21 binds to proliferating cell nuclear antigen (PCNA) and prevents it from activation of DNA polymerase, an activity required for DNA replication and repair. It plays an important role in regulating the proliferation, apoptosis, differentiation [5–7], metastasis [8] and stemness of cancer cells [9]. The losses of both expression and topological regulation of p21 is commonly detected in colorectal cancer [10]. Several observations implicated that cell cycle arrest and apoptosis of colon tumor cells could be stimulated by the induction of p21 [5, 11, 12]. Positive p21 expression has been suggested as an indicator for good prognosis in patients with colorectal cancer [7]. Therefore, activation of p21 is a promising way to suppress colorectal cancer.

Small RNAs (sRNAs) are an abundant class of small non-coding RNAs that could regulate diverse developmental and physiological processes via multiple mechanisms [13]. In the past decades, RNA interference (RNAi: guided by siRNAs known as small interfering RNA) is known as revolutionary form of therapy on cancer treatment by degrading homologous mRNA molecules or inhibiting protein translation [14–16]. Recently, literatures reported a sRNA-induced gene expression activation phenomenon known as RNAa, which is a small double strand RNA and termed as small activating RNA or saRNA, could activate sequence-specific gene transcription by targeting gene promoter regions [17, 18]. It appears that saRNAs could provide specific effect with minimal toxicity, similar to that of siRNA; moreover, the gene-regulatory effect by saRNA was profounder and longer than that by siRNA [17–20], because the epigenetic change induced by saRNA is presumably inheritable across cell generation [16]. Thus, saRNA, an emerging member of the sRNA world, might shed new light on our understanding of complex RNA networks and act as a promising therapeutic strategy for cancer [21].

Since Li et al. synthesized p21-saRNA-322 [17], research has been focused on its tumor suppressing activity and demonstrated that up-regulating p21 via p21-saRNA-322 led to inhabitation of cell growth in human cancer cell lines including bladder [22, 23], prostate [24], lung [25], liver [26], kidney [27] and glioma [28], *in vitro* and *in vivo*. However, to bring the p21-saRNA-322 into drug R&D, a proper drug delivery system has become a key issue. It appears that colorectal cancer could be a potential target for the p21-saRNA-322, as p21 down-regulation is a signature abnormality of the cancer and in rectal application might be a suitable approach for p21-saRNA-322 delivery.

The goal of the present study is to learn the activity of p21-saRNA-322 in colorectal cancer. We investigated the ability of p21-sa-RNA-332 to activate p21 expression and its effect on colorectal cancer cell lines, as well as in xenograft colorectal tumor mice. In what presented below we show that p21-saRNA-332 might be a promising alternative way in the treatment of colorectal cancer.

## RESULTS

### The p21-saRNA-322 stimulated p21 expression in the colorectal cancer cells

**Figure 1:**
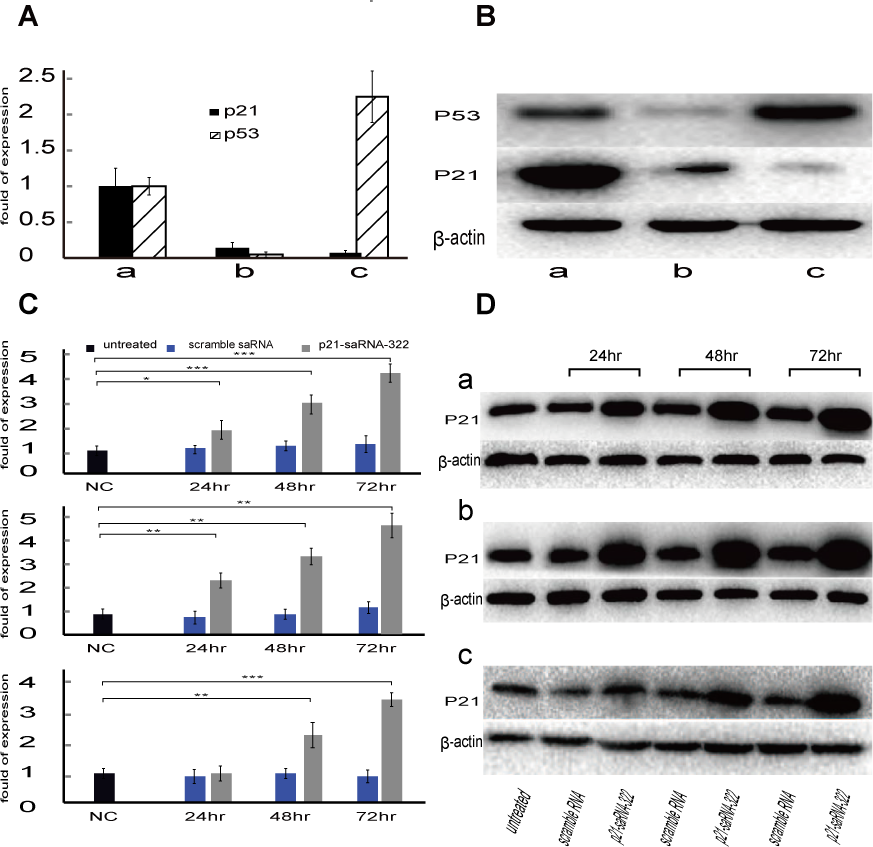
The p21-saRNA-322 activates p21 gene expression in colorectal carcinoma cell lines (**A**) The p21 and p53 mRNA expression of three colorectal cancer cell lines were detected by RT-PCR. The results were presented as means ± SD of three independent experiments and normalized to GAPDH. (**B**) The P21 and P53 protein expression was detected by Western blot analysis. The results were normalized to β-actin as density ratio. Human colorectal cancer cell lines, (a) HCT-116, (b) HCT-116 (p53−/−), and (c) HT-29, were treated with 25nM saRNA, scramble RNA as well as the untreated were used as references for 24, 48 or 72hrs, respectively. (**C**) Induction of p21 mRNA expression was analyzed by RT-PCR. The results were presented as means ± SD of three independent experiments and normalized to GAPDH. Expression levels were measured as fold relative to that of untreated reference. (**D**) Induction of P21 protein expression was detected by Western blot. The results were normalized to β-actin. Note exposure time for HT-29 cells was prolonged to 120s instead of 20s for HCT-116 in order to make a relatively even presentation of WB signal bands. Statistical significance is indicated as * (*p* < 0.05) and ** (*p* < 0.01), comparing to that of untreated group.

Three well-characterized human colorectal carcinoma cell lines (HCT-116, HCT-116 (p53−/−) and HT-29) were chosen to study the ability of p21-sa-RNA-332 to activate p21 expression. Before transfection, the levels of p21 and p53 in the three cell lines were determined at both the mRNA and protein levels. As shown in Figure 1A, HT-29 possessed the lowest expression of p21 (less than 5% of that in HCT-116), while the p53 expression was deprived in HCT-116 (p53−/−) (less than 5% of that in HT-29). Western-blotting analysis showed results similar to that of mRNA expression (Figure 1B).

The p21-saRNA-322 was used to activate p21 expression, in which HCT-116, HCT-116 (p53−/−) and HT-29 cells were transfected with 25 nM of p21-saRNA-322 using scramble RNA as well as the untreated as references. 24, 48 and 72 hrs later, the expression of p21 mRNA was tested by RT-PCR and P21 protein level was analyzed with Western blot. As shown in Figure 1C and Figure 1D, compared to the reference groups, the expression of p21 in p21-saRNA-322 treated cells was significantly elevated in all of the three cell lines. For HCT-116 and HCT-116 (p53−/−) cell lines, p21-saRNA-322 transfection caused 2.0- and 2.4-fold, 3.0- and 3.3-fold, 4.1- and 4.5-fold increase in mRNA, respectively, 24, 48 and 72 hrs after transfection. The elevation of p21 mRNA expression in HT-29 cell lines was seen 48 hrs after transfection, and the delay could be explained by its longer doubling time in respect to that of the HCT-116 and HCT-116 (p53−/−) cell lines (Figure 1C). Western blot analysis showed similar results in three cell lines (Figure 1D).

### Suppressing effects of p21-saRNA-322 on colorectal cancer cell growth

Then, we investigated the effects of p21-saRNA-322 activated p21 expression on colorectal cancer cells.

### The p21-saRNA-322 caused cell cycle arrest at G_0_/G_1_ phase in colorectal cancer cells

**Figure 2:**
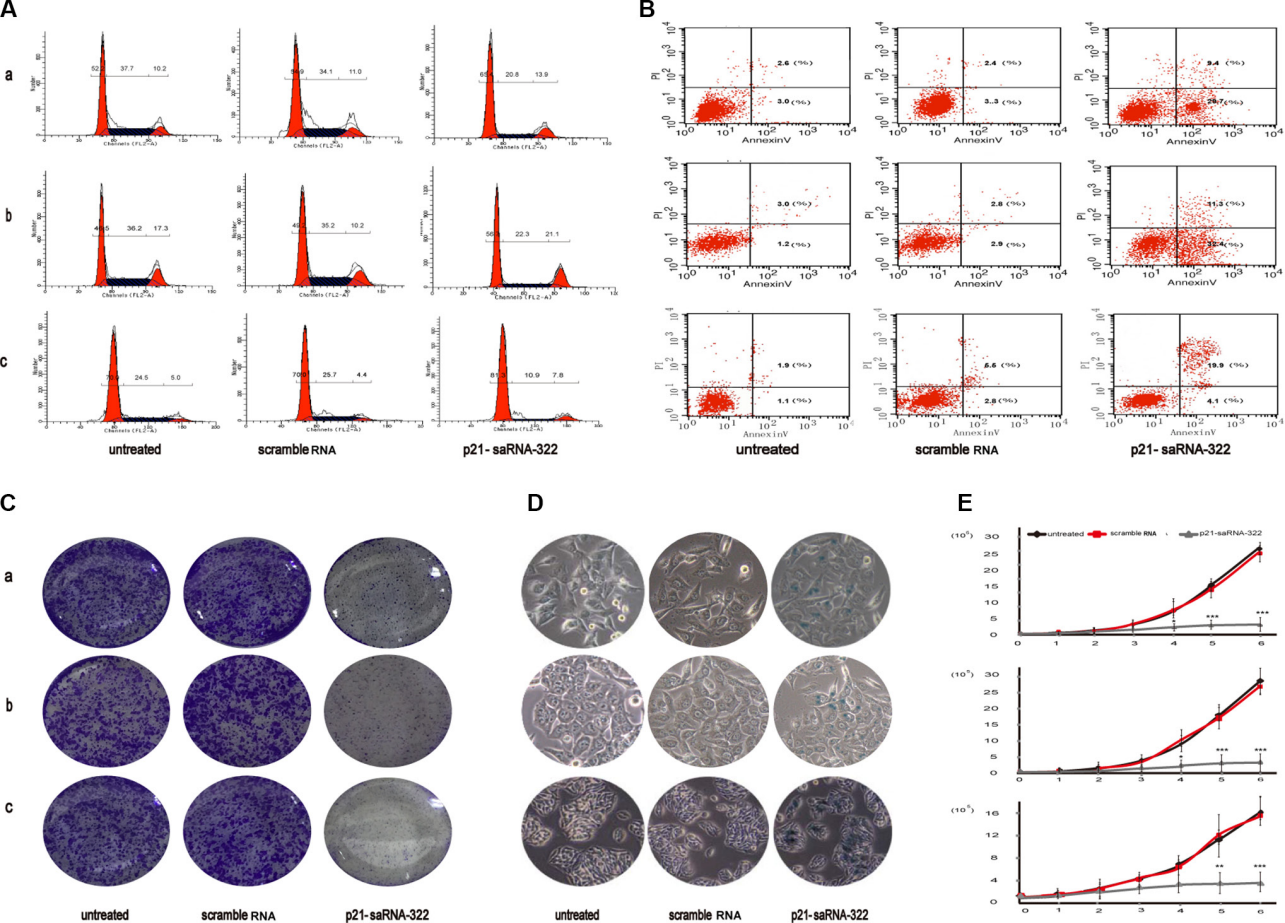
Effects of p21-saRNA-322 on colorectal cancer cells. Colorectal cell lines: HCT-116 (a), HCT-116 (p53−/−) (b) or HT-29 (c) was transfected with p21-saRNA-322 at 25 nM for 48 hrs, scramble RNA and untreated cells were used as negative reference (**A**) Shown is representative graph indicating cell distribution in the G_0_/G_1_, S and G_2_/M phases. Activation of p21 by p21-saRNA-322 causes cell cycle arrest of HCT-116, HCT-116 (p53−/−) and HT-29 cells at G_1_/G_0_. (**B**)The p21-saRNA-322 induced cells apoptosis in the colorectal cancer cells. Shown is the representative flow cytometry image of cell apoptosis. Annexin V-stained cells represent the early apoptotic cells; Annexin V+ propidiumiodide-stained cells demonstrate the late apoptotic cells. (**C**) The p21-saRNA-322 suppressed colony formation in colorectal cancer cells. Colony formation was tested by staining cells with crystal violet solution, shown are representative photographs taken from each treatment group. (**D**) The transfection of p21-saRNA-322 induces cell senescence in colorectal cancer cells. Cellular senescence was measured by β-galactosidase assay. Shown are representative of cell senescence. The p21-saRNA-322 transfected cells were positive for SA-b gal, evidenced by cytoplasmic blue color staining. SA-b gal activity: the blue color. (**E**) The p21-saRNA-322 suppressed cell proliferation in colorectal cancer cells. Cell proliferation was determined by cell counting on a daily basis. Each time point data represents the mean ± standard deviation of six independent experiments. Cells of reference groups showed an exponential growth, whereas the growth of the cells with p21-saRNA-322 transfection was markedly suppressed.

In the present study, we investigate the influence of cell cycle distribution by p21 activation via p21-saRNA-322 in human colorectal cancer cells using flow cytometric analysis. As shown in Figure 2A, the percentage of the cells in the G_0_/G_1_ phase was increased in the p21-saRNA-322 treated group (65.4% for HCT-116, 56.7% for HCT-116 (p53−/−) and 81.3% for HT29), as compared to that in the mock group (52.2% for HCT-116, 46.5% for HCT-116 (p53−/−) and 70.0% for HT29) and the scrambled RNA treated group (54.9, 49.2 and 70.0%, respectively for the three cell lines). The transfection with p21-saRNA-322 also respectively caused decrease in the S phase cells (20.8% for HCT-116, 22.3% for HCT-116 (p53−/−) and 10.9% for HT29 in p21-saRNA-322 group, in the comparison to those in the mock group: 37.7, 36.2 and 25.7%, and the scrambled RNA treated group: 34.1, 35.2 and 25.7%, respectively in the three cell lines), suggesting a cell cycle arrest at the G_0_/G_1_ checkpoint. These results are in agreement with previous studies [30].

### The p21-saRNA-322 induced apoptosis in the colorectal cancer cells

A large body of literature indicated that p21 was an important apoptosis protein in colorectal cancer [32–35]. Therefore, the induction of cancer cell apoptosis was evaluated by flow-cytometric analysis, in which colorectal cells were labeled with PI and Annexin V. As shown in Figure 2B, p21-saRNA-322 introduction significantly increased the proportion of apoptotic cells at both early and late stage in all of the three colorectal cancer cell lines. By 48 hrs after transfected with p21-saRNA-322, 32.1, 43.7 and 24.0% of the cells were respectively in apoptotic phase, as compared to the mock group (5.6, 4.2 and 3.0%) and the scramble RNA group (5.7, 5.7 and 8.3%,) in the HCT-116, HCT-116 (p53−/−) and HT-29 cell lines respectively.

### The p21-saRNA-322 inhibited cell proliferation and colony formation

Accumulative research proved that, p21 induction is a determinant in the regulation of colorectal cancer cells proliferation [20, 36]. In this study, we investigated whether p21-saRNA-322 transfection was responsible for cell proliferation and colony formation suppression. Our results showed that induction of p21 expression significantly inhibited cell proliferation and colony formation of colorectal cancer cells. As shown in Figure 2C, the cells transfected with p21-saRNA-322 showed significantly lower colony formation, compared with reference groups. The cell count detection via TC 20TM Automated Cell Counter (Bio Rad, CA, USA) was used to assess the proliferation ability of colorectal cancer cells for 6 days after transfection. As shown in Figure 2E, cells in reference groups showed an exponential growth, whereas the growth was markedly suppressed by p21-saRNA-322 transfection and the growth arrest was observed 2 days after transfection.

### The p21-saRNA-322 induced colorectal cancer cell senescence

A group of evidence demonstrates that p21 is required for cellular senescence [33, 38–40]. To evaluate the effect of p21 activation by saRNAs on cell senescence, we tested senescence-associated β-galactosidase expression in the colorectal cancer cells. As shown in Figure 2D, p21-saRNA-322 transfected cancer cells expressed intensive SA-β-gal activity, which is well known of being a marker of senescence, whereas, little or no SA-β-gal activities were detected in the cells of reference groups. These results suggested that the p21-saRNA-322 transfection might cause a senescence-like transform in human colorectal cancer cells.

### Inhibition effect of p21-saRNA-322 on colorectal tumor progression in nude mouse xenograft

**Figure 3:**
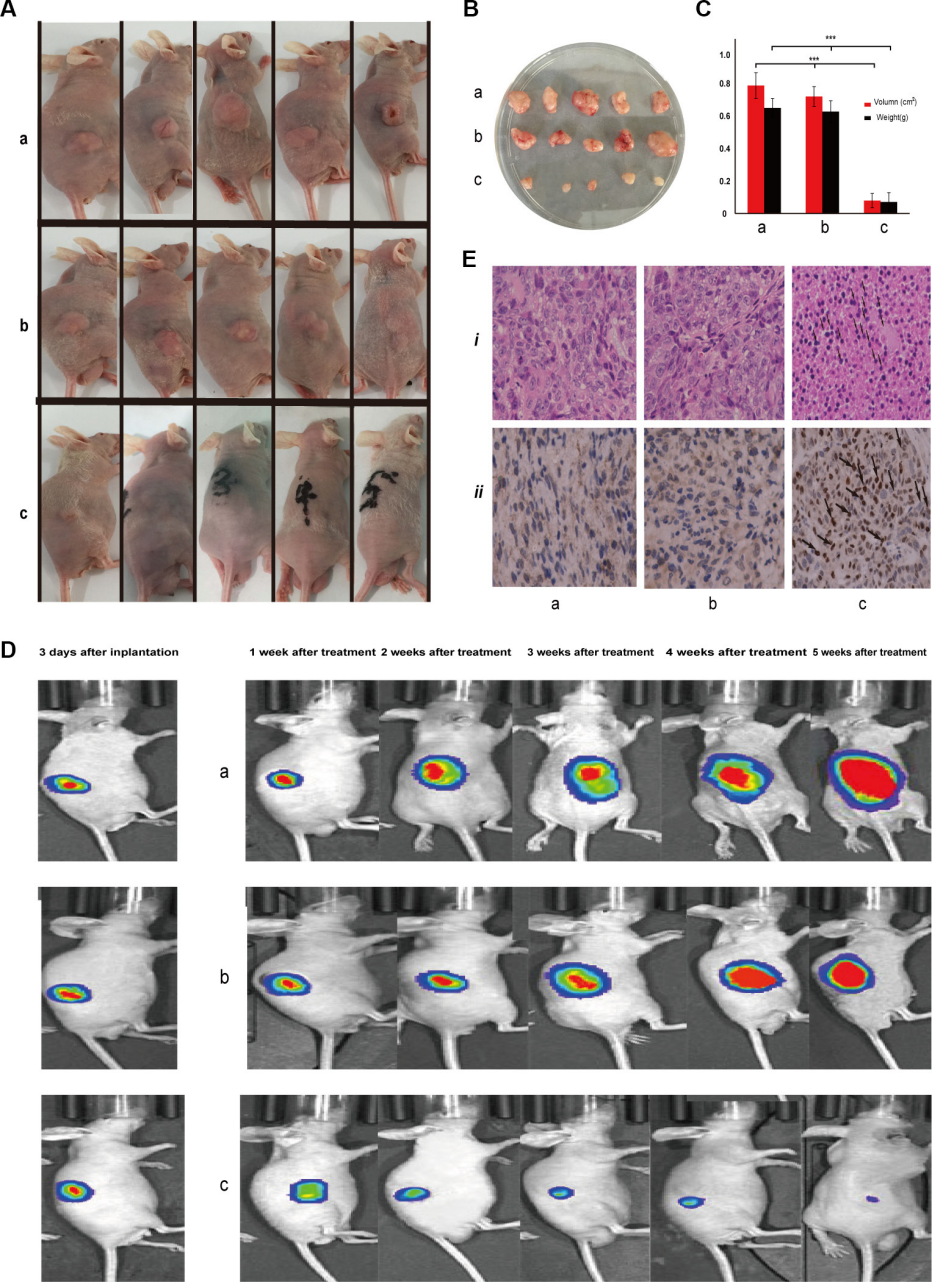
Intratumoral delivery of the P21-saRNA-322-containing Lipotamine inhibited subcutaneous colorectal tumor growth. P21-saRNA-322 (c) was injected intratumorally every 3 days for 5 weeks. Scramble RNA (b) and PBS (a) were used as reference (**A**) Representative photograph of host mice with subcutaneous tumors. (**B**) Representative photograph of tumor mass. (**C**) Presented are tumor volume and weight. (**D**) Shown are BLI images of a representative mouse of the each treatment group at the indicated time points (days). Cancer growth was monitored by evaluating subcutaneous tumor bioluminescence over 5 weeks of each animal. (**E**) Representative H&E staining (i) and immunohistochemical staining (ii) of p21 in tumor slides.

To determine whether the stimulation of p21 expression by p21-saRNA-322 suppresses tumor progression *in vivo*, HT-29-Red Fluc colorectal cancer cells were injected subcutaneously into nude mice. Bioluminescence signal of tumor growth was measured by testing photons/second following intraperitoneal administration of luciferase substrate. The HT-29-Red Fluc colorectal tumor-bearing mice were randomly divided into three groups, phosphate buffer saline (PBS), scramble RNA and p21-saRNA-322 groups. No statistical difference in signal intensity or body weight was detected among groups at time of randomization. Three days after tumor implantation, intratumoral injection (of PBS, or scramble RNA, or P21-saRNA-322) was applied every three days for 5 weeks. Bioluminescence signal was measured every week to monitor the growth of tumor mass. At the end of the study, all of the mice were sacrificed and tumors were surgically removed. Representative photographs of the host mice and the corresponding tumor masses are indicated in Figure 3A, 3B. Tumor volume and weight were also demonstrated (Figure 3C). The median tumor volumes for PBS, scramble RNA, and P21-saRNA-322 treatment groups were 0.79 cm^3^, 0.72 cm^3^, and 0.13 cm^3^, respectively; the median weights were 0.61, 0.63 and 0.10 g, respectively. The results revealed that, p21-saRNA-322 treatment significantly reduced the tumor burden, whereas no significant difference was detected in the 2 reference groups. Tumor bioluminescence within each mouse was monitored longitudinally and plotted over 5 weeks for all treatment groups. As shown in Figure 3D, the intensity of tumor bioluminescence in PBS and scramble RNA treatment groups increased with time, whereas bioluminescence intensity in animals treated with p21-saRNA-322 gradually decreased. These results demonstrate that, p21-saRNA-322 could effectively suppress colorectal tumor growth in a nude mouse xenograft and is consistent with the research of dsP21-322 lipidoid-based nanoparticles on regression of prostate xenograft tumors and bladder orthotopic tumors [17, 24].

Immuno-histochemistry (IHC) test revealed that, cells positive for p21 were very rare and randomly dispersed in the tumor tissue of the control groups, while the p21-saRNA-322-treated xenograft tissue were symbolized with intense nuclear staining of p21 (Figure 3E). The result suggests that intratumoral administration of p21-saRNA-322p21 activated p21 expression *in vivo*, which seemed to be the mechanism responsible for the tumor growth inhibition.

## DISCUSSION

In the present work, we explored the therapeutic potential of p21-saRNA-322 on human colorectal cancer. Three well-characterized human colorectal carcinoma cell lines HCT-116, HCT-116 (p53−/−) and HT-29 were chosen based on their dissimilarity in p53 and p21 gene expression. Among the three cell lines, the lowest expression of p21 was observed in HT29 cell line and the p53 expression was deprived in HCT-116 (p53−/−) cells. Based on that, we investigated whether the introduction of p21-saRNA-322 could activate p21 gene expression in the colorectal cancer cells, and whether the activity of p21-saRNA-322 was influenced by intrinsic p21 expression and p53 integrity. We found that induction of p21 gene was quite robust to all of the three colorectal cancer cell lines, and importantly, for p53 negative ones. Moreover, the elevated p21 expression was detectable for over 10–15 days after p21-saRNA-322 transfection, longer than siRNA induced gene expression change [14–16]. This phenomenon was probably because that, the mechanism of saRNA was targeting gene’s promoter regions, inducing demethylation of histones, altering the chromatin structure and leading to prolonged and profound augmentation of gene expression [14–16]. It is our assumption that p21-saRNA-322 might be efficient in its action against cancer due to its long-term p21 expression induction. The consequence of p21 up-regulation by p21-saRNA-322 was cell cycle arrest, cell growth inhibition, cell apoptosis, colony formation suppression and cellular senescence. The therapeutic potential of p21-saRNA322 on colorectal cancer was evidenced in the human xenograft tumors following intratumoral injection.

The major effect of p21-saRNA-322 is to directly up-regulate p21 expression. p21 is a multifunctional cyclin-dependent kinases (CDK) inhibitor (CKI), which suppressed tumorigenesis by inhibiting cell cycle progression [3]. p21 is particularly important in the tumor-genesis of colorectal cancer. Accumulating research showed that p21 played an important role in blocking cell cycling [29–31], inhibiting cell proliferation [5, 20, 36], inducing cell apoptosis [6, 32–35], regulating cell differentiation and leading to cellular senescence [33, 38–40] in human colorectal cancer. The losses of both expression and topological regulation of p21 is commonly detected in colorectal cancer [10, 41]. Several observations implicated that cell cycle arrest and apoptosis of colon tumor cells could be stimulated by the induction of p21 [5, 11, 12]. Positive p21 expression has been suggested to be an indicator for good prognosis in patients with colorectal cancer [7]. Therefore, p21 is a promising target to treat colorectal cancer [20]. However, p21 gene is a client protein of p53, the most frequently mutated cancer suppressor gene in human cancers [6]. p21 activation partially depends on the expression and function of p53. Liu and Bodmer found that 76.8% (43 in 56) of the studied colorectal cancer cell lines had p53 mutation and almost half of the mutations were in truncating form [42]. Park et al., reported that, p21 activation induced by extracellular signal-regulated kinease (ERK) pathway was lost in the HCT-116 (p53−/−) cells, thus HCT-116 (p53−/−) was found to be insensitive to some anticancer agents [20]. It’s crucial to find a direct way of stimulating p53-independent activation of p21 expression.

Recently, literatures reported a small active RNA (saRNA)-induced gene expression activation phenomenon known as RNA activation (RNAa). Distinguished from small interference RNA (siRNA), specific small double strand RNA (termed as small activating RNA or saRNA) could directly activate target gene transcription through binding specific gene promoter regions [17], recruiting enzymes such as methyltransferase, and then inducing demethylation of histones. Since the RNAa alters the chromatin structure and causes robust and prolonged expression of the endogenous target gene, it represents an efficient approach in the treatment of cancer by specifically activating silenced genes or augmenting the expression of tumor suppressor genes expressed at low level. Therefore, the p21-saRNA-322, which selectively activated p21 gene expression, caused accumulating attention. Researches proved that p21-saRNA-322 could suppress cell growth in human cancer cell lines including bladder, prostate, lung, liver, kidney and glioma, *in vitro* and *in vivo* via arresting cell cycle inhibiting cell proliferation and colony formation, and inducing cell apoptosis as well as cellular senescence. Result for the activity of p21-saRNA-322 on CRC has not been reported yet.

saRNA induced gene activation represents a novel approach for the treatment of human cancer; however, RNAa-based clinical research has been restricted due to the susceptibility of RNA molecules to serum nucleases, renal clearance and non-specific bio-distribution. In addition, potential activation of innate immune system by nuclear acids could lead to unexpected toxicities and undesirable effects [43, 44]. Studies suggest that *in situ* drug delivery might overcome those obstacles. The advantages of the *in situ* delivery are focused-delivery of RNA into target tumor tissue, decreased adverse events as well as reduced immune reactions. Several local delivery strategies for therapeutic RNA have been developed, including intracranial [45], intraocular [46] and intravesical treatment [47]. Naked siRNA-based treatments applied locally to the disease site have been in clinical trials [48]. Kang et al confirmed that saRNA possesses anti-proliferation activity in bladder cancer by facilitating p21 induction, and intravesical treatment of mice with orthotopic bladder cancer inhibited tumor growth and extended animal survival [23]. Our previous work showed that, direct rectal drug delivery was an efficient way to treat colorectal cancer [49, 50]. Thus, it is justified to investigate the effect of p21-saRNA-322 on colorectal cancer in the *in-situ* administration route. In the present study, we have successfully verified the cancer suppressing effect of p21-saRNA-322 on xenograft animal model. Based on that, our next attempt is to develop a clinically relevant orthotopic animal model to evaluate the therapeutic effect of p21-saRNA-322 using rectal administration technique. We consider it an important step toward possible future application in human.

## MATERIALS AND METHODS

### Reagents

The saRNAs (dsp21-322: S, 5′-CCA ACU CAU UCU CCA AGU A [dT][dT]-3′; AS, 5′-U ACU UGG AGA AUG AGU UGG [dT][dT]-3′ and negative control: S, 5′-UUC UCC GAA CGU GUC ACG U [dT][dT]-3′; AS, 5′-ACG UGA CAC GUU CGG AGA ATT-3′) were designed as described by Li et al [46] and chemically synthesized by GenePharma (Suzhou, China).

### Cell culture, and transfection

The human CRC cell line HCT-116 and HT-29 were abstained from China Infrastructure of Cell line Resources (Beijing, China). HCT-116 (p53−/−) cells (chosen as deficiency of p53) was kindly gifted by Ph. D Zhan from cancer hospital *CAMS*. HT-29 was cultured in McCoy’s 5A (Modified), HEPES medium supplemented with 10% (v/v) fetal bovine serum (FBS), penicillin (100 U/mL), and streptomycin (100 μg/mL). HCT-116 and HCT-116 (p53−/−) were maintained in RPMI1640 supplemented with 10%FBS, 100 U/ml of penicillin, and 100 ug/ml of streptomycin. All cell lines were cultured in a humidified atmosphere containing 5% CO_2_ and maintained at 37°C.

The day before transfection, HCT-116, HCT-116 (p53−/−) cells were seeded into 12-well plates with antibiotics-free growth medium at a density of 4–5 × 10^4^ cells/well and cultured overnight to 10–20% confluence. For HT-29, the original density was 1–1.5×10^5^ cells/well. Transfections of p21-saRNA-322 and scramble RNA were carried out at a concentration of 25 nM/ well using Lipofectamine 3000 reagent (Invitrogen, CA, USA) according to the manufacturer’s protocol and lasted for 24, 48 and 72 hrs.

### Real-time quantitative reverse-transcriptase polymerase chain reaction (RT-PCR)

Cells were collected at 24, 48 and 72 hrs after treating with 25 nM p21-saRNA-322, scramble RNA treated as well as the untreated cells were used as references, rinsed twice with ice-cold PBS. Total RNA was extracted using TRIzol^®^ Plus RNA Purification Kit (Invitrogen, CA, USA). The concentration of RNA was measured by spectrophotometer ND2000 (Thermo Scientific, MA, USA). 5ng of total RNA was used for quantitative RT-PCR, which was performed on the Applied Biosystems 7500 Fast Real-Time PCR System (Applied Biosystems, CA, USA) using Power SYBR^®^ Green RNA-to-CT™ 1-Step Kit (Applied Biosystems, CA, USA). PCR amplification included an initial denaturation step (95°C for 10 min), 40 cycles of denaturation (95°C for 10 s), and annealing (60°C for 1 min). Values are showed as fold-differences compared to that of untreated reference group. Expressions were normalized to glyceraldehyde-3-phosphate dehydrogenase (GAPDH).

The p21 PCR primer sequences: 5′-cttcga ctttgtcaccgaga-3′ (forward), 5′-ggtccacatggtcttcctct-3′ (reverse); GAPDH PCR primer sequences: 5′-AGAACA TCATCCCTGCCTCT-3′ (forward), 5′-CTGCTTCACCAC CTTCTTGA-3′ (reverse). All experiments were done in triplicate and independently validated three times.

### Western blotting analysis

Cells were collected at 24, 48 and 72 hrs after treatment, rinsed twice with ice-cold PBS, harvested, lysed in Pierce RIPA buffer (Thermo Scientific, MA, USA). Cell lysates were clarified by centrifugation at 12000 × g for 30 min at 4°C and protein concentrations were determined using the BCA protein assay reagent (Pierce, MA, USA). Cell lysates (protein concentration was adjusted by adding certain amount of RIPA buffer) were subjected to 12% sodium dodecyl sulfate-polyacrylamide electophoresis (SDS-PAGE) gels, separated by SDS-PAGE and electrophoretically transferred to Invitrolon polyvinylidene difluoride (PVDF) membranes (Life technologies, CA, USA). Membranes were blocked with 5% skim milk and then incubated overnight with the appropriate primary antibodies (Seajet Scientific Inc, Beijing, China) followed by matching horseradish peroxidase-conjugated secondary antibodies. After washing each sample three times for 10 min with 15 ml TBST, immobilon Western Chemiluminescent HRP Substrate (Millipore, MA, USA) was added and the result was tested with ChemiDoc XRS^+^ electrophoretic imaging system (Bio-Rad Laboratories, Berkeley, USA).

### Cell cycle analysis

Flow cytometry assay was used to analyze cell cycle. HCT-116, HCT-116 (−/−) and HT-29 cells were plated onto the 6-well plate at the density of 1 × 10^5^, 1 × 10^5^ and 2 × 10^5^ cell/plate, respectively. After cultured for 24 hrs, cells were transfected with 25 nM p21-saRNA-322 using Lipofectamine 3000. Scramble RNA treated and untreated cells were used as reference. 48 hrs after treatment, cells were harvest, rinsed by PBS twice and fixed in (80%) cold ethanol at 4°C overnight. Fixed cells was collected by washing with PBS twice and centrifuged at 300 × g for 5 min. 500 μL of Prodium Iodide (PI) staining solution (50 mg/ml PI, 10 mg/ml RNAse A, 0.1% Triton X-100, and 0.1% sodium citrate in PBS) were added into each tube, incubated for 30min at 37°C in the darkness. The stained cells were immediately analyzed at BD FACSCalibur (BD Biosciences, CA, USA). Distribution of cell cycle was showed as the percentage of cells in G_0_/G_1_, G_2_/M and S populations.

### Apoptosis analysis

Apoptosis of colorectal cancer cells were assessed by flow cytometry after co-staining of cells with Annexin V-FITC and PI in accordance with manufacturer’s recommendations (BD Biosciences, CA, USA). Annexin V FITC positive, PI negative cells and double-positive cells were considered to be in early and late stages of apoptosis, respectively [26].

### Cell proliferation

The cell count detection via TC 20TM Automated Cell Counter (Bio Rad) was performed to assess the effect of p21-saRNA-322 on cell proliferation. HCT-116, HCT-116 (p53−/−) and HT-29 Cells were transfected with 25 nM p21-saRNA-322 using Lipofectamine 3000 for 12 hrs, scramble RNA treated cells as well as the untreated cells were used as references. Following treatments, cells were plated in 12-well plates at a density of 1 × 10^4^ cells (5 × 10^4^ cells for HT-29 cells) in 1 mL of complete cell culture medium per well for proliferation assay. Every 24 hrs for the following 6 days, a batch of cells were collected and the cell number was detected using TC 20TM Automated Cell Counter (Bio Rad). All experiments were performed in six duplicate.

### Colony formation assay

As described by wang et al with little modulation [26], HCT-116, HCT-116 (p53−/−) and HT-29 Cells were transfected with 25 nM p21-saRNA-322 using Lipofectamine3000 for 12 hrs, scramble RNA treated cells as well as the untreated cells were used as references. Following treatments, cells were transferred to six-well plates and seeded at a density of approximately 1.0 × 10^3^ cells per well. Culture medium was changed every 3 days. Colony formation was analyzed 12 days after treatment by staining cells with a 0.05% crystal violet solution for 1 hr.

### Cell senscence

Described by Phalke et al [39], senescence was assessed by a senescence-associated β-galactosidase assay (Beyotime technology, Beijing, China). In brief, Cells were harvested 48 hrs after treatment, washed twice with ice cold PBS, fixed with 0.2% gluteraldehyde and 2% formaldehyde for 5 min at room temperature. Collected cells were incubated with SA-β-gal staining solution (1 mg/ml X-gal, 2 mM MgCl_2_, 5 mM each of potasium ferricynide and potassium ferrocynide in 40 mM citric acid/sodium phosphate buffer) for 2–8 hrs at 37°C in dark. After incubation, the cells were washed with PBS and images were captured using an Olympus (Olmpus, Tokyo, Japan) inverted light microscope. The experiment was conducted triplicate and counting was done on three randomly selected fields each plate.

### *In vivo* anti tumor activity of p21-saRNA-322

Six- to eight-week-old male nude mice were purchased from Beijing Weitong Lihua Experimental Animal Technology Co. Ltd (Beijing. China). All animals in this study were housed under pathogen-free conditions and were maintained in accordance with the guidelines of the recommendations for the Care and Use of Laboratory Animals of the National Institutes of Health. The protocol was approved by the Committee of the Ethics of Animal Experiments of the Chinese Academy of Medical Sciences & Peking Union Medical College. 3 × 10^5^ Bioware Brite HT29-Red-Fluc (Perkin, MA, USA) colorectal cancer cells suspended in 50 uL PBS was injected subcutaneously into the right posterior limb. Three days following tumor cell implantation, *in vivo* bioluminescence imaging of HT29-Red-Fluc cells was evaluated on a IVIS Spectrum Imaging System (PerkinElmer, MA, USA) following intraperitoneal administration of 200 uL D-luciferin (15 mg/kg) (PerkinElmer, MA, USA), Bioluminescence signal was measured using Living Image^®^ 4.3.1. Regions of interest were defined to quantify signal intensity. The mice were randomized to three groups. Group 1 (five mice each) received intratumoral injection of Lipofectamine3000-encapsulated p21-saRNA-322 (30 ug/mouse) every three days for 5 weeks beginning at day 4. Group 2 and 3 (five mice each) were injected intratumorally with Lipofectamine3000-encapsulated scramble RNA (30ug/mouse) or Lipofectamine3000-mixed PBS every three days for 5 weeks beginning at day 4. Bioluminescence signals were monitored weekly. The study was terminated 5weeks following treatment. All mice were subsequently euthanized and tumors surgically removed, the volume of the tumor was calculated with the formula: V = (width^2^ × length^0.5^) and tumor weight was assessed. The tumor tissue was placed in 10% neutral buffered formalin for immune histochemistry (IHC). Results are expressed as means ± SD.

### Statistical analysis

Data are expressed as mean ± standard deviation (SD). Statistical analysis of data was done with Student’s *t-test*, using SPSS15.0 Sigma Plot software (St. Louis, MO, USA). The correlation between variables was analyzed using Pearson correlation coefficient and *p* < 0.05 was considered to be a statistically significant difference.

## CONCLUSIONS

The present study demonstrated that introduction of p21-saRNA-322 significantly increased p21 expression in colorectal cancer cells, which in turn generated negative biological effects on colorectal cancer cell growth. To confirm the role of p21-saRNA-322 *in vivo*, we extended the research into the nude mouse xenograft model, and found that the p21-saRNA-322 *in situ* administration significantly inhibited xenograft growth. These results indicate that the strategy of treating colorectal cancer via p21-saRNA-322 merits further investigation.
